# Mutations in Distal Cis-Regulatory Elements in the *Beta Globin* (*HBB*) Gene Promoter of Filipinos Influences Transcriptional Activity

**DOI:** 10.3390/genes17091055

**Published:** 2026-08-31

**Authors:** Yousef Abdulrahman E. Lawal, Norman C. Garvida, Christian Adam L. Espiritu, James Patrick K. Peralta, Ebner Bon G. Maceda, Jimmbeth Zenila P. Fabia, Peter James Icalia Gann

**Affiliations:** 1 Center for Cellular and Molecular Medical Research, College of Medicine, Mariano Marcos State University, Batac 2906, Ilocos Norte, Philippines; youseflawal@gmail.com (Y.A.E.L.); jpkperalta@mmsu.edu.ph (J.P.K.P.); zpfabia@mmsu.edu.ph (J.Z.P.F.); 2 Department of Biology, College of Arts and Sciences, Mariano Marcos State University, Batac 2906, Ilocos Norte, Philippines; 3 Center for Research and Development, Davao Medical School Foundation, Inc., Davao City 8000, Davao del Sur, Philippines; caespiritu@email.dmsf.edu.ph; 4 Department of Biochemistry and Molecular Biology, College of Medicine, Mariano Marcos State University, Batac 2906, Ilocos Norte, Philippines; 5 Department of Medical Microbiology, College of Medicine, Mariano Marcos State University, Batac 2906, Ilocos Norte, Philippines; 6 Institute of Human Genetics, National Institutes of Health, University of the Philippines Manila, Manila 1000, Philippines; egmaceda@up.edu.ph

**Keywords:** beta-thalassemia, *beta-globin* gene, hemoglobinopathies, promoter, transcriptional regulation

## Abstract

**Background/Objectives**: Hemoglobin is a component of the red blood cell responsible for carrying oxygen. It is composed of four chains, two of each for the alpha and beta globin. Mutations in the gene, *beta-globin* (*HBB*), coding for the beta-globin chains causes beta-thalassemia. Common diagnostic methods for this genetic disorder cover only the coding region of the *HBB* gene. **Methods**: Cis-regulatory elements within the distal *HBB* promoter region were predicted in silico. Promoter variants were identified through PCR amplification and Sanger sequencing, while *HBB* gene expression was assessed. Hematologic parameters were measured, and associations between promoter variants, gene expression, and hematologic parameters were statistically analyzed. **Results**: Sequencing showed varying mutations in the predicted cis-regulatory elements which suggests less binding of transcription factors leading to a potential change in *HBB* gene activity. This is corroborated by the gene expression analysis where the presence of more mutations in the promoter region resulted in a higher downregulation of the *HBB* gene which manifested in hematologic analysis as lower mean corpuscular volume (MCV) and hemoglobin (HGB). Among the cis-regulatory element mutations that potentially explain the reduced expression in *HBB* are binding to transcription factors such as TATA and GATA. **Conclusions**: Our work illuminates the importance of expanding analysis of beta-globin variations to the promoter region for understanding the causes of thalassemia traits that cannot be explained by conventional diagnostic methods to employ appropriate treatment strategies.

## 1. Introduction

Hemoglobin (Hb), the main protein in red blood cells, carries oxygen throughout the body. Adult hemoglobin (Hb A) consists of two alpha and two beta globin chains, produced by alpha-globin-1/alpha-globin-2 (*HBA1*/*HBA2)* gene (chromosome 16) and *beta-globin* (*HBB)* gene (chromosome 11) genes [1]. Mutations in *HBB* genes or their regulatory regions can reduce or halt globin chain production, leading to beta-thalassemia, a disorder marked by microcytic hypochromic anemia and impaired oxygen transport [2,3,4,5]. Current diagnostic tools for beta-thalassemia include capillary electrophoresis and fragment sequencing [6,7,8], which covers only the structural region of the *HBB*. The promoter region which regulates the activity of the *HBB* and may contribute to beta-thalassemia trait is not covered by existing diagnostic methods.

The *HBB* promoter contains several conserved cis-regulatory elements which are essential for proper transcriptional regulation of the *HBB* gene [9,10,11,12]. Numerous Single Nucleotide Polymorphisms (SNPs) have been identified in the *alpha-globin* and *beta-globin* gene promoters [13,14] and numerous other studies, some of which influence promoter activity, gene regulation, and disease susceptibility [15,16]. Many functional mutations cluster between +50 to −500 from the transcription start site (TSS), though others occur further upstream [17]. Distinguishing between polymorphic and pathogenic variants is crucial for accurate clinical interpretation [13].

There were previously reported mutations in the promoter region of *HBB* gene. Cis-regulatory elements described include CACCC boxes, CCAAT box, TATA box which were both hypothesized to result in beta-thalassemia or even “silent” thalassemia. A study in the Spanish population reported mutations that were distributed across key regulatory elements of the *HBB* gene promoter, with one variant in the distal CACCC box, two in the proximal CACCC box, and three in the ATA box causing moderate beta-thalassemia, and there were also silent mutations where carriers have normal blood parameter indices [16]. In the Chinese population, carriers of a novel −73 (A → T) CCAAT-box had a completely silent β-thalassemia phenotype despite one compound heterozygote child showing microcytosis [18]. In another study where ~192,000 individuals were screened, a novel 5′-UTR (cap region) mutation c.23A > G was found with 75 carriers (≈0.0389%) but there were no noticeable hematologic parameters between carriers and controls [19].

Additionally, other studies have likewise identified single-nucleotide substitutions upstream of the TSS in individuals exhibiting normal or borderline hematological parameters, including red blood cell (RBC) count, mean corpuscular volume (MCV), mean corpuscular hemoglobin (MCH) and hemoglobin (HGB) [20,21,22]. Unlike coding-region substitutions that alter amino acid residues, these promoter variants occur within regulatory elements and primarily affect the transcriptional activity of the *HBB* gene. These mutations showing a hematologic picture without microcytosis and only borderline for other β^+^thalassemia indicators make routine screening ineffective. Moreover, these findings reinforce that promoter variants in *HBB* gene promoter can produce very mild or silent effects on blood indices. These mutations in the regulatory region of the *HBB* gene can be overlooked in conventional diagnosis which poses risks with possibility of additive impact from independent assortment.

This study was conducted to evaluate the potential of mutations within the *HBB* gene promoter by analyzing associated *HBA2* levels, *HBB* gene expression profiles and complete blood count (CBC) parameters among participants. Moreover, the identification of potentially novel promoter variants may provide additional evidence supporting the clinical relevance of regulatory-region mutations in β-thalassemia. The findings of this study may contribute to improving current diagnostic strategies by emphasizing the importance of including the *HBB* promoter region in routine molecular screening for thalassemia, particularly in the cases of mild, borderline, and silent hematologic phenotypes. Taking into account the aims of this study and the availability of resources, the analysis focused only on detecting mutations within the distal region of the *HBB* gene; comparisons of promoter transcriptional activity between mutations and wild-type (WT) sequences are not covered. Furthermore, mutations located in the untranslated regions (UTRs), intronic, and exonic regions were not analyzed, and the *HBA1* and *HBA2* genes were not included in the scope of this research.

## 2. Materials and Methods

### 2.1. Study Design and Participant Recruitment

This cross-sectional, laboratory-based study investigated the role of distal cis-regulatory elements in the *HBB* gene promoter and their influence on transcriptional activity. Based on geographic proximity and feasibility, participants were recruited from Filipino faculty, student, and staff of Mariano Marcos State University (MMSU), Batac, Ilocos Norte, using snowball sampling over a 2-week recruitment period between 1 April and 14 April 2026. The short timeframe allotted for participant recruitment may have limited the number of eligible participants included in the study. Study information was disseminated through posters, and written informed consent was obtained prior to participation. Eligible individuals were assigned unique identification numbers, and participants were selected using a random number generator. Inclusion criteria were based on age (>18 years old with no upper age limit) and had not previously undergone thalassemia screening, while individuals diagnosed with thalassemia or other hemoglobinopathies, laboratory evidence or clinical suspicion of iron deficiency, active severe infection or advanced medical illness affecting hematologic parameters or recent transfusions within the past 3 months were excluded. The study protocol was approved by the institutional ethics review board [PN: 2025-538].

### 2.2. Demographic and Hematologic Data Collection

Demographic information, including age and sex, was collected using standardized data collection forms. Additional variables not part of the inclusion and exclusion criteria, such as BMI/BRI, alcohol use history, and smoking history, were not collected in this study. Peripheral blood samples were obtained via venipuncture and collected in ethylenediaminetetraacetic acid (EDTA) tubes (DISPOLAB, Troubsko, Czech Republic). Hematologic parameters, including HGB, RBC, MCV, and MCH, were measured using Biobase Auto Hematology Analyzer (MN: BK-3200; BIOBASE Group, Jinan, Shandong, China) following manufacturer protocols.

### 2.3. Genomic DNA Extraction and Quality Assessment

Genomic DNA was extracted from peripheral blood leukocytes using GF-1 Tissue/Blood DNA Extraction Kit (Cat No. GF-BT-100; Vivantis Technologies Sdn. Bhd., kuala Lumpur, Malaysia), following the manufacturer’s protocol. DNA concentration and purity were assessed using spectrophotometric analysis by measuring absorbance ratios at 260/280 nm (Analytik jena Scandrop^2^ SN: 3830A-0376; Analytik Jena GmbH+Co. KG, Jena, Germany). DNA integrity was further evaluated through agarose gel electrophoresis.

### 2.4. Identification of Cis-Regulatory Elements in the HBB Promoter

The upstream regulatory region of the *HBB* gene, including distal promoter elements, was extracted from NCBI database (RefSeq Accession number: NC_000011.10; Gene ID: 3043). In silico analysis using the retrieved sequence was conducted to predict cis-regulatory elements and transcription factor binding sites (TFBSs) using the JAPSAR CORE Database. Emphasis was placed on identifying distal regulatory motifs, including enhancer- and silencer-associated sequences. Motif scanning and positional analysis were performed to map potential TFBSs within the promoter region.

### 2.5. PCR Amplification and Sequencing of the HBB Promoter Region

Primers targeting distal regions of the *HBB* promoter were designed using NCBI Primer-BLAST. Polymerase chain reaction (PCR) amplification was carried out in a thermal cycler under optimized conditions. Amplified products were verified by agarose gel electrophoresis and subsequently purified. Purified PCR products were subjected to Sanger sequencing. Capillary sequencing involves the incorporation of fluorescently labeled chain terminator ddNTPs. The reaction components include amplicons, corresponding primers, and the ABI BigDye^®^ Terminator v3.1 Cycle Sequencing Kit (Cat No. 4337455; Applied Biosystems™, Thermo Fisher Scientific, Foster City, CA, USA). The cycling parameters on the thermal cycler were as follows: pre-hold at 4 °C; 96 °C for 1 min; 25 cycles at 96 °C for 10 s, 50 °C for 5 s, 62 °C for 4 min; and hold at 4 °C. Ethanol precipitation was performed to remove unincorporated ddNTPs, excess primers, and primer dimers. Capillary electrophoresis was carried out on the ABI 3730 xl DNA Analyzer using a 50 cm 96-capillary array, POP7 Polymer (Cat No. 4393714; Applied Biosystems™, Thermo Fisher Scientific, Foster City, CA, USA), and 3730 xl Data Collection Software v3.1. Lastly, base calling was performed using the Sequencing Analysis Software v5.4.

### 2.6. Assessment of HBB Transcriptional Activity

Total RNA was extracted from peripheral blood reticulocytes using a phenol–chloroform-based method. Complementary DNA (cDNA) was synthesized via reverse transcription using the PrimeScript™ RT Reagent Kit (Cat. No. RR037A; Takara Bio Inc., Kusatsu, Shiga, Japan). Quantitative real-time PCR (qPCR) was performed using gene-specific primers on an Applied Biosystems QuantStudio™ 5 system (SN: 275210988; Applied Biosystems™, Thermo Fisher Scientific, Foster City, CA, USA) with TB Green^®^ Premix Ex Taq™ II (Cat. No. RR820; Takara Bio Inc., Kusatsu, Shiga, Japan). Gene expression levels were normalized to the housekeeping gene *ACTB* [23,24], and relative expression was calculated using the 2^−ΔΔCt^ method.

### 2.7. Transcription Factor Binding Analysis

Sequence alignment of participant-derived sequences with the reference sequence obtained from NCBI (RefSeq Accession number: NC_000011.10; Gene ID: 3043) was performed using EMBL-EBI online Clustal Omega to evaluate sequence variation. Predicted TFBS profiles of the reference and participant sequences were subsequently compared to assess the potential impact of these variants on transcription factor binding using Unipro UGENE software (Version No. 53.1).

### 2.8. Statistical Analysis

Descriptive statistics were used to summarize demographic and hematologic data. Differences between groups were assessed using independent samples *t*-tests. When comparisons involved more than two groups, one-way analysis of variance (ANOVA) was performed followed by Tukey’s post hoc multiple comparison test to determine pairwise differences. A *p*-value of <0.05 was considered statistically significant.

## 3. Results

### 3.1. Demographic of Participants

A total of 10 Filipino participants were included in the study. The majority were females (60%, *n* = 6), while males comprised 40% (*n* = 4). In terms of age, 50% of the participants were within the 21–30 years age group, followed by 31–40 years (40%) and 41–50 years (10%). The ages ranged from 22 to 41 years old with a mean age of 32 ± 6.78 years.

### 3.2. Predicted Cis-Regulatory Elements in the HBB Gene Promoter

**Figure 1:**
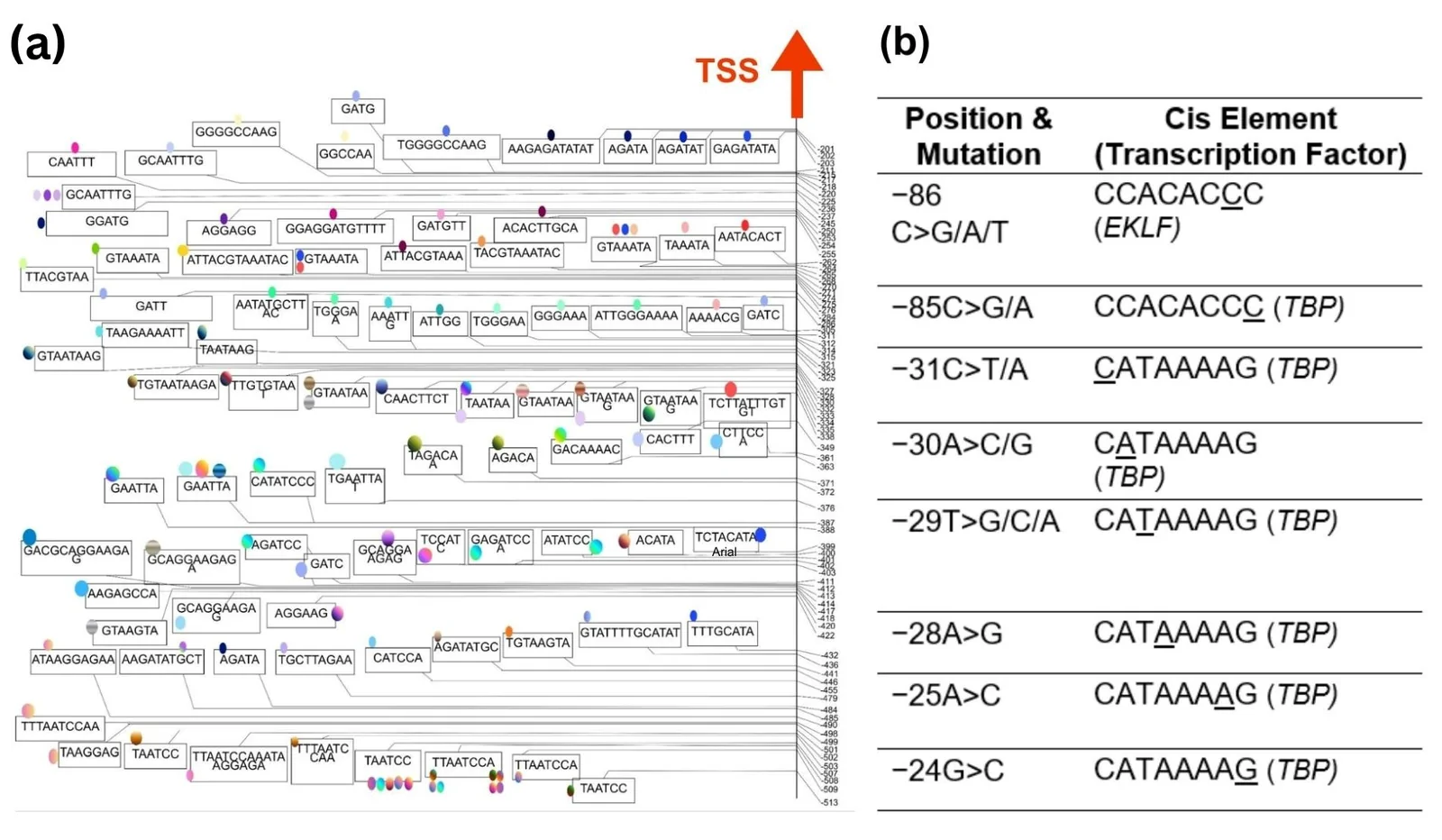
Predicted cis-regulatory elements and previously reported mutations in the promoter region in the *HBB* gene. (**a**) Diagram of the distal region of the promoter of *HBB* gene covering −200 to −750 bp from the TSS showing the predicted cis-regulatory elements and the potentially binding transcription factors. The arrow represents the location of the TSS with respect to the given positions. Different colored circles represent the transcription factors. Complete analysis of the predicted cis-regulatory elements and their associated transcription factors in the *HBB* promoter is presented in the Supplementary Figure S1 and Supplementary Table S1. (**b**) Mutations reported in the proximal region of the promoter of *HBB* gene covering the −100 to −1 bp from the TSS. Data in this table were curated from the HbVar database [25].

The promoter region of the *HBB* gene was analyzed to identify putative cis-regulatory elements and the previously reported mutations in the *HBB* promoter were summarized (Figure 1a,b). A total of 300 TFBSs were detected within the −750 bp to −200 bp region upstream of the TSS, corresponding to a high density of regulatory elements across the promoter region (Figure 1a). Among these, a few were recognized to be associated with erythroid differentiation and globin gene regulation. These included the GATA-binding motifs which were located at multiple positions. Interestingly, the multiple high-scoring GATA-binding motifs clustered between −750 bp to −200 bp suggest potential regulatory regions crucial for erythroid-specific activation. Other significant TFBSs included those for transcription factors SPI1, FL1 and ELF1, respectively. These regulatory elements were distributed across the distal regions of the promoter. The details of these TFBSs are presented in Supplementary Table S1 and Supplementary Figure S1.

Beyond the said region, a total of 14 CACC box motifs were found to be clustered within the −110 bp to −70 bp region including an additional isolated CACC motif located at −912 bp, supporting their known role as binding sites for Krüppel-like factors (KLFs) (Figure 1b). In addition, two MYB TFBSs were identified downstream of the TSS. Further upstream (beyond −750), five GATA motifs, one ETS1 binding site and four SPI binding motifs were also predicted. These elements were relatively dispersed along the distal promoter region rather than forming tight clusters.

Moreover, a diverse set of predicted TFBSs corresponding to non-erythroid regulatory proteins were also identified within the *HBB* gene promoter. These included members of several transcription families such as the forkhead box (FOX), homeobox (HOX), Basic Leucine Zipper (bZIP), Basic Helix–Loop–Helix (bHLH), Zinc Finger (ZNF) Proteins and others.

Furthermore, several of these TFBSs were found to coincide or occur in close proximity to one another, forming clusters of regulatory motifs within specific regions in both upstream and downstream segments of the promoter. High relative scores for several predicted motifs support the likelihood of true biological significance, although experimental validation is necessary.

### 3.3. Hematologic Profile and Transcriptional Activity of HBB Gene

#### 3.3.1. Differential CBC Parameters Across Participants

**Figure 2:**
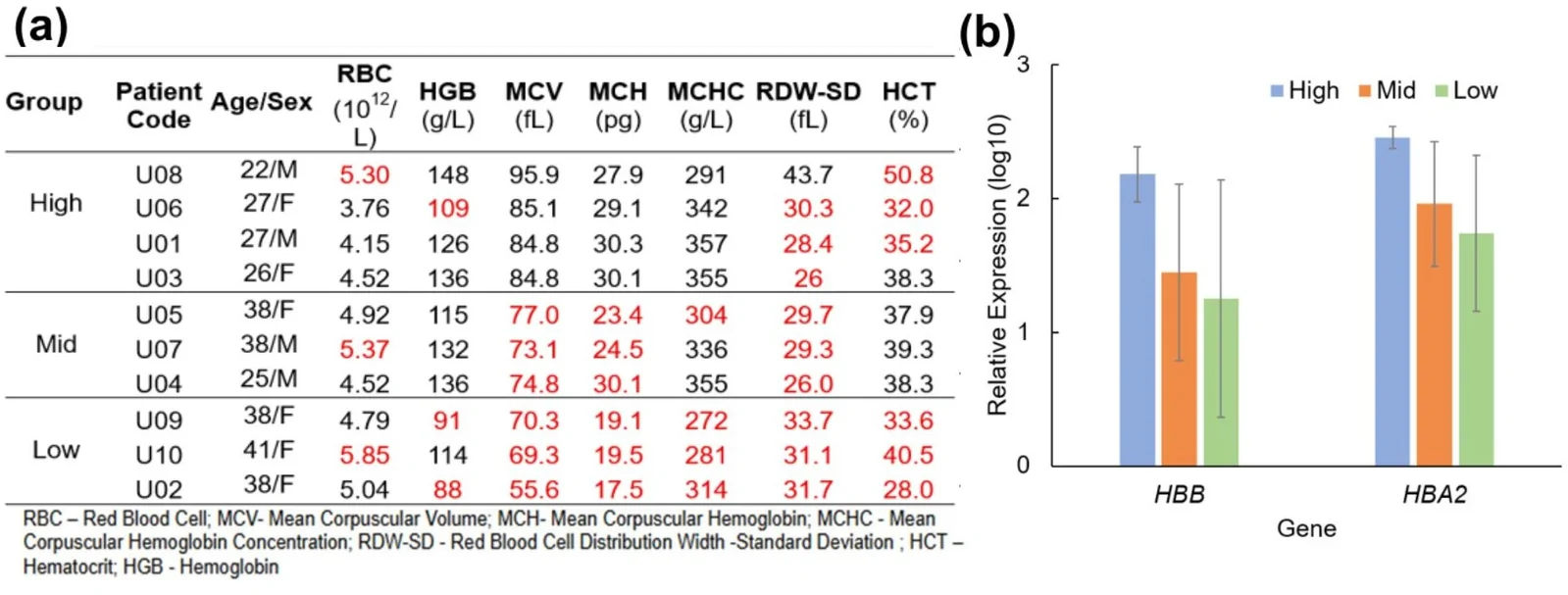
Complete blood count (CBC), demographics and expression of *HBB* and *HBA2* gene among the participants. (**a**) MCV and HGB of participants is variable with groups falling with and outside normal range. Values highlighted in red are not within normal range. Reference ranges are RBC (3.5–5), HGB (110–150), MCV (80–100), MCH (27–34), MCHC (320–360), RDW-SD (35–56), HCT (37–47). CBCs with all the parameters are presented in Supplementary Table S2. (**b**) There is differential regulation of *HBB* and *HBA2* across groups of participants based on MCV (x ≥ 80 y = 71–79, z ≤ 70). Data presented in bars are mean ± standard deviation. There were two technical replicates in each participant and error bars indicate the standard error.

The hematologic profiles of the participants were assessed using standard CBC parameters and the pattern of *HBB* expression was evaluated (Figure 2a,b). Means were compared using Tukey’s multiple comparison at α= 0.05. and significant for condition as source of variation at *p* ≤ 0.01. A few participants exhibited reduced MCV and MCH values, while others had values within normal ranges (Figure 2a). Among the ten participants, the mean Hb level was 121.3 g/L (range: 88–148 g/L), while the mean RBC count was 4.88 × 10^6^/µL (range: 3.76–5.85 × 10^6^/µL). The mean MCV and MCH values were 77.89 fL and 25.14 pg, ranging from 55 fL and 17.5 pg to 95.9 fL and 27.9 pg, respectively. The mean hematocrit was 37.8% (range: 28–50.8%). The mean MCHC was 321.4 g/L (range: 272–362 g/L), while RDW-SD values averaged 31.18 fL (range: 26–43.7 fL). Based on the clinical severity of microcytosis, participants were categorized into three MCV groups: high MCV (≥80 fL; normocytic), mid MCV (71–79 fL; mild microcytosis), and low MCV (≤70 fL; severe microcytosis). This classification was based on the established definition of microcytosis as an MCV < 80 fL, with lower MCV values reflecting increasing severity of microcytosis [26]. Five participants (50%) belonged to the high-MCV group, two (20%) to the mid-MCV group, and three (30%) to the low-MCV group. Statistically significant differences in MCV were observed between the high and mid MCV groups (*p* < 0.05), and between the moderate and low MCV groups (*p* < 0.01). For MCH, significant differences were observed between the high and mid MCV groups (*p* < 0.01), as well as between the mid and low MCV groups (*p* < 0.01). Moreover, no statistically significant differences were observed in the other hematologic parameters.

Participants with lower MCV values generally manifested reduced MCH levels, while the values of other parameters showed variability across the study population. The individual hematologic parameters are summarized in Figure 2a and Supplementary Table S2.

#### 3.3.2. Differential Expression of *HBB* Gene and *HBA2* Gene Across MCV-Defined Groups

Relative expression analysis revealed differential regulation of globin genes across MCV-stratified groups (Figure 2b). Asterisk indicates significant differences between groups within a specific time period (* significant at α = 0.05 and ** significant at α = and the analysis of variance (ANOVA) table is presented in Supplementary Table S3.) *HBA2* exhibited the highest expression levels in both the high and mid MCV groups, with an abrupt decrease observed in the low group. A less pronounced progressive pattern was noticed in *HBB*, which showed a slight decreasing trend from high to low groups, whereas *HBA2* displayed greater variability and a wider range in expression levels.

Expression data were derived from two technical replicates per sample and presented as mean ± standard deviation, where expression values are shown on a log 10 scale. Tukey’s post hoc test identified significant differences among groups (*p* < 0.01). In terms of *HBA2* expression, there was no significant difference between the mid and high MCV groups (*p* > 0.01). However, both the mid and high MCV groups showed statistically significant differences when compared with the low MCV group (*p* < 0.01). This observation may be attributable to mutations in the *HBA2* gene. On the other hand, *HBB* expression levels showed less pronounced variation across the groups, but a slightly higher expression was observed in the high MCV group compared to the other groups. However, these differences were relatively small in magnitude, and no statistically significant differences were detected between the MCV groups.

### 3.4. Mutations in the HBB Gene Promoter

**Figure 3:**
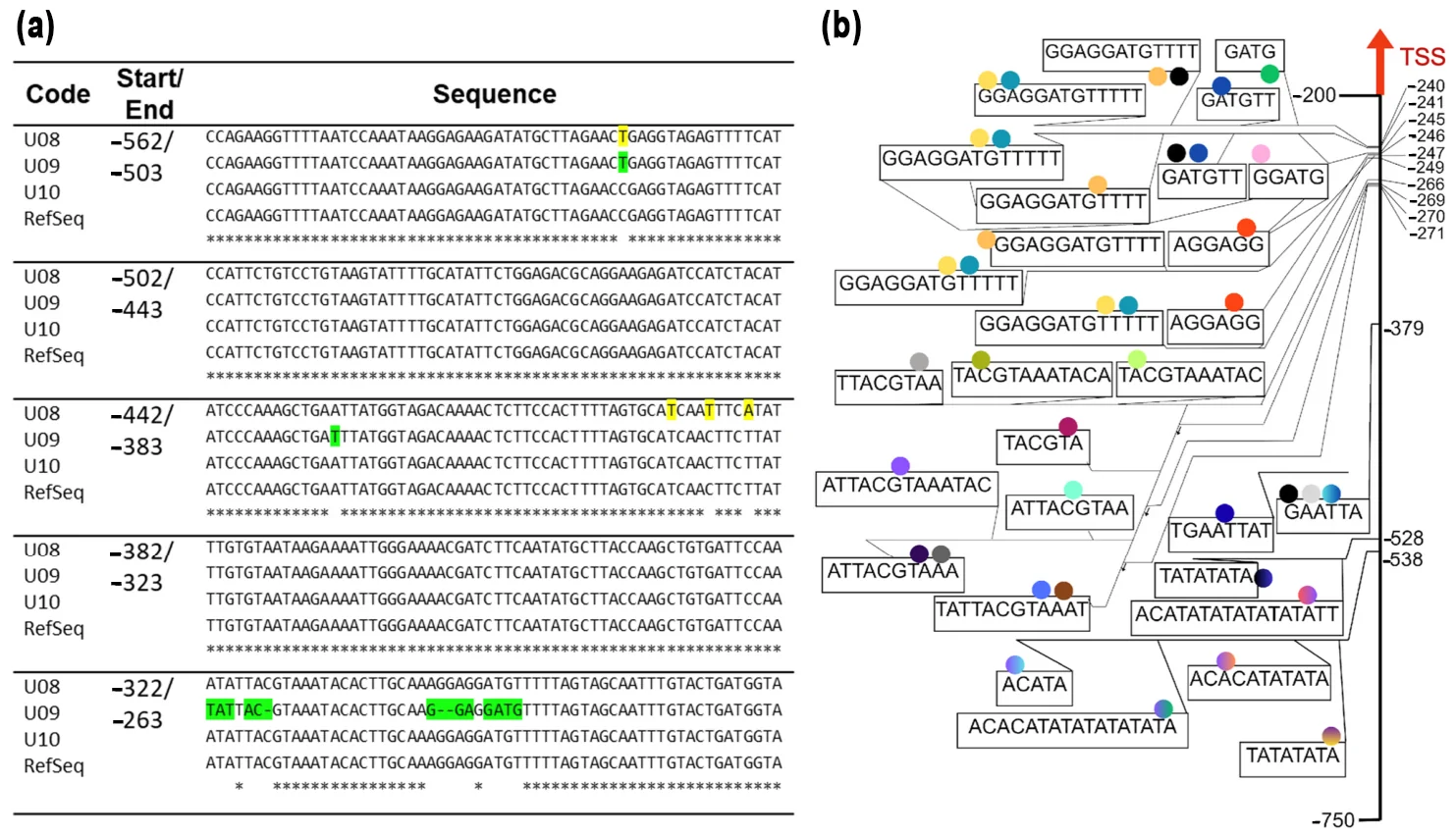
Mutations in the *HBB* gene promoter and analysis of the cis-regulatory elements affected by the mutations in the promoter. (**a**) Sequence alignment of the gene *HBB gene* promoter showing variants relative to the reference sequence across the participants. Representative sequences with the best chromatogram quality were selected for analysis. Asterisk (*) under the alignment shows similarity and gaps indicate mutation/s. Each highlighted color represents the mutation/s found in the same participant. The complete sequence alignment is presented in Supplementary Table S4 and chromatograms from Sangers sequencing are shown in Supplementary Figure S2. (**b**) A representative analysis of mutations within cis-regulatory elements and their predicted transcription factor (TF) binding sites in participant U09. The arrow represents the location of the TSS with respect to the given positions. Analysis of the all the mutations in cis-regulatory elements and corresponding TFs are shown in Supplementary Table S5 and Supplementary Figure S3.

A total of 25 sequence variations in two out of three participants were identified in the *HBB* gene promoter (Figure 3a). The identified mutations consisted of insertions, deletions, and primarily of single nucleotide substitutions, at different positions relative to the TSS. The identified mutations were located at various regions including −703, −551, −528, −340, −336, among others. However, the distribution of sequence variations varied across the promoter. Multiple mutations were observed within specific regions, particularly between −270 and −240 bp within the upstream segment thus indicating clustering of mutations.

In terms of mutation types, single nucleotide substitutions were repeatedly observed. Insertions and deletions were less common but were identified in specific regions of the promoter. Notably, an insertion of TA between positions −538 and −537 bp was observed in two participants (U08 and U09), implying a shared variant within this region. Moreover, three consecutive deletional mutations at positions −249, −248 and −247 bp were identified in participant U09.

At the participant level, participant U09 exhibited a greater number of mutations compared to U08, including a cluster of mutations within the −270 bp to −240 bp region as mentioned earlier. Conversely, participant U08 revealed fewer variants, including three heterozygous nucleotide substitutions at positions −703, −528 and −340 bp relative to the TSS. The detailed characteristics of the identified mutations, including their positions, and distributions across participants are summarized in supplementary Table S4.

### 3.5. Transcription Factors Affected by the Mutations

The identified mutations within the *HBB* gene promoter were analyzed to determine their potential impact on predicted TFBSs, with 22 variants found to overlap with these regulatory regions (Figure 3a). Several variants occurred within motifs essential for erythroid regulation, notably in participants U08 and U09. Specifically, participant U08 exhibited a heterozygous T > C mutation at position −703 relative to the TSS, which overlapped with predicted binding sites for the GATA2 and GATA6 transcription factors. Furthermore, participant U09 presented a cluster of four sequence variations at positions −245 G > A, −243 A > G, −242 T > A, and −241 G > T, all of which intersected with a predicted GATA2 binding motif (Figure 3b).

In addition to those linked to erythroid-specific regulation, the study identified sequence variations within predicted binding motifs for several non-erythroid transcription factor families, such as FOX, ZNF, and *HOX*. These mutations were distributed across the promoter’s upstream region, with each variant corresponding to a site where the binding affinity was likely altered, as indicated by relative scores exceeding 90%. By disrupting these diverse motifs, these variants may influence *HBB* regulation through mechanisms that extend beyond standard erythroid-associated pathways.

## 4. Discussion

This study provides evidence that sequence variation within distal cis-regulatory elements of the *HBB* promoter contributes to modulation of transcriptional activity and is reflected in measurable hematologic variation. While current diagnostic strategies primarily focus on coding mutations within the *HBB* gene, the present findings underscore the functional relevance of promoter-region variation, particularly within the transcription factor binding domain, in influencing the b-globin expression [1,27].

The in silico analysis revealed a dense distribution of TFBSs across the −750 to −200 bp upstream region, supporting the role of distal promoter elements as an extended regulatory domain, The clustering of GATA motifs within this interval is consistent with the established function of GATA transcription factors in erythroid differentiation and *HBB* gene activation [28,29]. In addition, the presence of CACCC boxes, SPL1, and MYB binding sites suggests that *HBB* transcription is governed by coordinated interactions among multiple erythroid regulators. Such combinatorial control mechanisms have been widely described as essential for precise temporal and tissue-specific expression of globin genes [27,30].

Sequencing of the promoter region identified multiple variants, several of which overlapped with predicted TFBSs. Variants located within GATA-binding motifs are of relevance, as disruption of these sites has been associated with reduced transcriptional efficiency through impaired transcription factor binding [31]. Similarly, alterations affecting CACCC elements may interfere with KLF-mediated activation of the *HBB* gene promoter. The observed clustering of mutations within specific promoter segments, particularly in the −270 to −240 bp regions, suggests the possibility of cumulative effects on transcriptional regulation, given that spatial organization of TFBSs can influence cooperative binding and transcriptional output.

Gene expression analysis demonstrated a decreasing trend in *HBB* gene expression across MCV defined groups, with lower expression observed in individuals with reduced MCV values. Although these differences did not reach statistical significance, the consistent directional pattern supports a potential regulatory effect of promoter variation. In contrast, *HBA2* expression exhibited a more pronounced reduction in the low MCV group, indicative of variability in globin gene regulation and suggesting possible compensatory mechanisms between a and *HBB* gene expression [32]. Disruption in the balance of globin chain synthesis remains a defining feature of thalassemia pathophysiology and may account for the hematologic patterns observed [5].

The hematologic data further support these molecular observations. Participants with lower MCV also demonstrated reduced MCH and MCHC, consistent with microcytic and hypochromic indices characteristic of beta-thalassemia traits. However, the presence of individuals with normal hematologic parameters highlights the subtle phenotypic impact of promoter variants. This aligns with previous reports describing mild beta-thalassemia phenotype associated with regulatory mutations, which often remain undetected in routine screening due to the absence of overt hematologic abnormalities [16,22].

The identification of promoter variants in individuals without clear diagnostics indicators emphasizes a limitation in current screening approaches. Standard molecular diagnostics typically exclude regulatory regions, thereby potentially overlooking variants that influence gene expression without altering the protein structure. The findings presented in this paper support the inclusion of promoter analysis in genetic screening frameworks, particularly in the cases of unexplained microcytosis and inconclusive hematological profiles [17,18].

In addition to erythroid-specific transcription factor binding sites, variants were also identified within the binding motifs of transcription factors not traditionally associated with globin regulation, including members of the FOX, *HOX*, and ZNF families. Although the functional implications of these interactions are not yet fully understood, their presence suggests that *HBB* gene promoter activity may be influenced by broader transcriptional networks, potentially through effects on chromatic accessibility [33,34].

The present study has several limitations that should be acknowledged. This is a pilot study conducted among MMSU staff and students, which means that findings are exploratory and are not generalizable to the Philippine population. The short timeframe allotted for participant recruitment also limited the number of eligible participants included in the study. Additionally, the scope of mutation analysis was restricted to the distal promoter region of the *HBB* gene; variants in the UTRs, intronic, and exonic regions, as well as the *HBA1* and *HBA2* genes, were not examined. Functional comparisons of promoter activity between identified mutations and WT sequences were also not performed.

The present study demonstrates that mutations within distal cis-regulatory elements of the *HBB* gene promoter are associated with layered transcriptional activity and subtle changes in hematologic parameters. Despite these limitations, the findings highlight the importance of regulatory regions in the genetic architecture of *beta-globin* gene expression and support the expansion of diagnostic strategies to include promoter analysis for improved detection and characterization of thalassemia-related phenotypes.

## Data Availability

All data generated or analyzed during this study are included in this published article.

## Supplementary Materials

The following supporting information can be downloaded at: https://drive.google.com/drive/folders/1be_G2kC0hB3XsPV6_h8XxKNOaCGEXCUT?usp=sharing, (accessed on 9 June 2026).

## Abbreviations

The following abbreviations are used in this manuscript:

ABI	Applied Biosystems
ACTB	Beta-actin
ANOVA	Analysis of Variance
bHLH	Basic Helix–Loop–Helix
bZIP	Basic Leucine Zipper
bp	Base Pair
CBC	Complete Blood Count
cDNA	Complementary DNA
ddNTPs	Dideoxynucleotide Triphosphates
DNA	Deoxyribonucleic Acid
EDTA	Ethylenediaminetetraacetic Acid
ELF1	E74 Like ETS Transcription Factor 1
ETS1	ETS Proto-Oncogene 1
FLI1	Friend Leukemia Integration 1 Transcription Factor
FOX	Forkhead Box
GATA	GATA Binding Protein
Hb	Hemoglobin
HBA1	Hemoglobin Subunit Alpha 1
HBA2	Hemoglobin Subunit Alpha 2
HBB	Hemoglobin Subunit Beta
HGB	Hemoglobin
*HOX*	Homeobox
JASPAR	Jaspar Database of Transcription Factor Binding Profiles
KLF	Krüppel-Like Factor
MCH	Mean Corpuscular Hemoglobin
MCHC	Mean Corpuscular Hemoglobin Concentration
MCV	Mean Corpuscular Volume
NCBI	National Center for Biotechnology Information
PCR	Polymerase Chain Reaction
qPCR	Quantitative Polymerase Chain Reaction
RBC	Red Blood Cell
RNA	Ribonucleic Acid
RT	Reverse Transcription
SNP	Single Nucleotide Polymorphism
SPI1	Spi-1 Proto-Oncogene
TFBS	Transcription Factor Binding Site
TSS	Transcription Start Site
UTR	Untranslated Region
ZNF	Zinc Finger Protein
